# Case Report: New genetic variant and widely different phenotypes observed in twins with Vanishing White Matter disease

**DOI:** 10.3389/fped.2025.1643040

**Published:** 2025-08-07

**Authors:** Ellis Oron-Lexner, Bjørk Ditlev Larsen, Maria Therese Schelde-Olesen

**Affiliations:** ^1^ Department of Pediatrics and Adolescent Medicine, Sygehus Sønderjylland, Aabenraa, Denmark; ^2^ Department of Clinical Genetics, Odense Universitetshospital, Odense, Denmark

**Keywords:** Vanishing White Matter disease, CACH VWM syndrome, eukaryotic initiation factor-2B, leukoencephalopathy, case report, phenotype

## Abstract

**Introduction:**

Vanishing White Matter disease (VWM) is a rare neurological disease, with an autosomal recessive inheritance. In this case report, we describe two four-year-old dizygotic twin brothers diagnosed with VWM with the same genotype and different phenotypes. We also describe a new likely pathogenic variant leading to VWM.

**Method:**

Clinical examination, radiologic analysis and genetic workups, including whole genome sequencing and trio-genome analysis were conducted to diagnose and describe the patients' disease.

**Results:**

A four-year-old boy was hospitalized with acute loss of motor functions and a somnolent state after a minor head trauma. Based on the clinical evaluation, radiological findings and genetic analysis he was diagnosed with VWM. The proband's twin carried identical pathogenic variants and exhibited white matter lesions on MRI. However, unlike the proband who presented with non-specific symptoms since the age of one, his twin remained asymptomatic at diagnosis.

**Discussion:**

This case may indicate that factors other than genotype could affect the age of onset of VWM. During the genetic analysis a previously unknown genetic variant was detected, which is now classified as a likely pathogenic variant of VWM.

## Introduction

Vanishing White Matter disease (VWM; OMIM: 603,896), also called Childhood Ataxia with Central nervous system Hypomyelination (CACH), is an autosomal recessive leukoencephalopathy with an estimated incidence from large genomic databases of 1 per 715.000 live births (1, 2).

VWM disease is characterized by progressive neurological deterioration, primarily cerebellar ataxia, spasticity and optic atrophy, together with episodes of acute worsening triggered by stressors. These stressors can be divided into physiological stressors such as fever, infections, minor head trauma, and psychological stressors such as acute fright. During these episodes, patients experience rapid loss of motor function and become hypotonic, which together with lowering of consciousness, can result in coma or death (3, 4).

The onset of Vanishing White Matter disease varies and spans from the antenatal period to late adulthood, but is most common in early childhood (3, 5).

The diagnosis of VWM disease is based on the clinical representation, MRI findings, and confirmed by genetic testing (6). MRI criteria are defined, but the characteristic findings are most commonly seen in patients with the classic early childhood onset VWM (3). VWM is caused by homozygote or compound heterozygote pathogenic variants in one of five genes (*EIF2B1, EIF2B2, EIF2B3, EIF2B4*, and *EIF2B5*), which encode the five subunits of the eukaryotic translation initiation factor 2B holocomplex (EIF2B) (5).

The genetic condition occurs when a child inherits a pathogenic copy of one of the genes from each parent. Both parents are in most cases healthy carriers and thereby have a 25% probability of conceiving a child with the disease. In some cases, one of the variants is *de novo* (6, 7).

No statistically significant phenotype correlation by gene has been observed regarding age of onset and survival. Age of onset is a strong predictor for the disease course and survival (7).

Considering the rarity of VWM disease the objective of this report is to describe two Danish Caucasian four-year-old dizygotic twin brothers diagnosed and genetically confirmed with VWM with the same genotype and different phenotypes. Furthermore, we describe a new likely pathogenic variant in the *EIF2B3* gene leading to VWM.

## Case

### Clinical evaluation

A four-year-old Caucasian male patient (proband and hereon referred to as Twin A) was hospitalized after a minor head trauma, which happened while playing in his kindergarten. This led to acute loss of motor functions and a somnolent state. At the time of hospitalization, the patient was already under neuropediatric investigation. Initially after admittance, the patient slowly woke up; however, his motor abilities remained impaired relative to the pre-trauma baseline.

**Figure 1:**
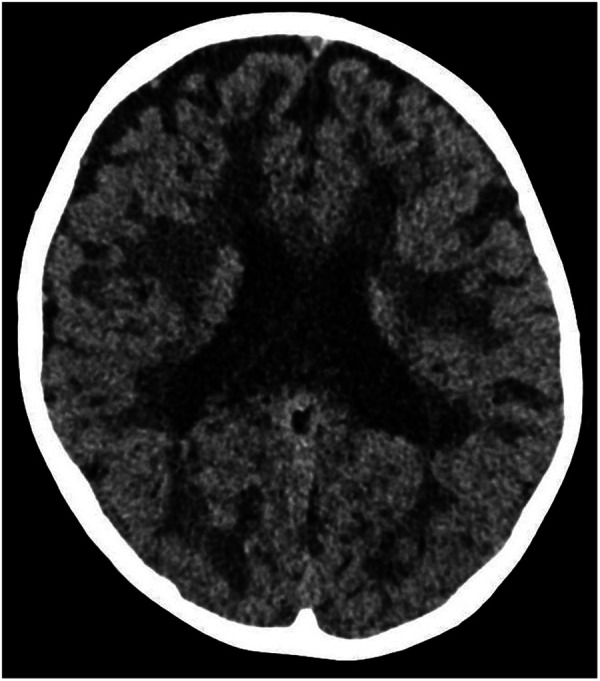
The emergency cranial CT scan of twin A. Axial scan shows white matter lesions, which raised the suspicion of VWM.

An emergency cranial CT scan (Figure 1), conducted shortly after arrival, showed changes in the white matter and it was concluded that VWM should be considered due to these findings.

The genetic analysis, received soon after the CT scan, found genetic variants in the *EIF2B3* gene, which could be linked to Leukoencephalopathy with VWM type 3. This supported the clinical and radiological findings while awaiting a MRI scan.

During the week of hospitalization, the patient regained some of his motor function skills. He could support his head, and use both arms and hands with a slight deficiency in the left side. Further improvement of the motor function skills was observed during the following months, however the patient could only walk with support and was mostly wheelchair-bound. It was further noted that the patient had increased ataxia after the head trauma.

The parents relayed that the patient had exhibited developmental delay and ataxia from around one year of age. The motor delay was even more apparent when comparing to his dizygotic male twin (hereon referred to as Twin B). The patient received physiotherapy initiated by his kindergarten. As worries persisted, the family was referred to the hospital at the age of 3.5 years, and clinical investigation was initiated. The parents further mentioned that the twins' birth was an uncomplicated vaginal birth, where the proband was born first. They also mentioned that Twin A was more frequently sick than his siblings and the disease duration were longer. In retrospect Twin A experienced acute motor function loss during fever or sickness. These losses were of short duration and the patient regained his baseline abilities after a few days.

As VWM age-of-onset varies, it was decided to genetically examine the patient's twin brother and older sibling. This analysis showed that Twin B had the same two variants as Twin A in the *EIF2B3* genes, while their older sibling did not carry any of the variations. Twin B did not at that time, display observable clinical symptoms of the disease. Furthermore, according to the parents, Twin B did not experience any loss of motor skills after sickness or fever episodes.

**Figure 2:**
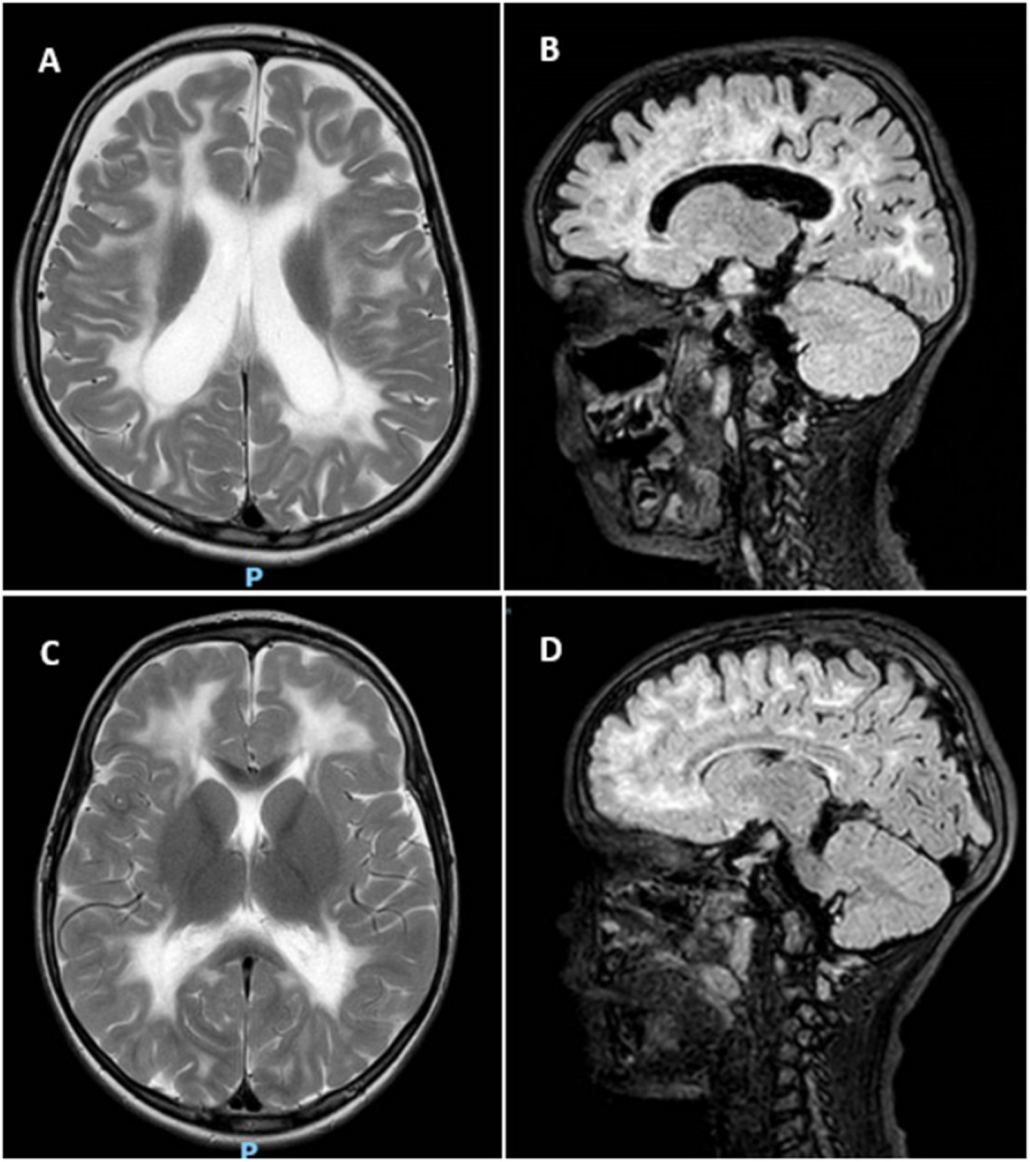
MRI of twin A and B. **(A)** T2 weighted axial scan of Twin A shows extensive cerebral white matter abnormalities with minimal sparing of subcortical white matter. The lateral ventricles are dilated due to white matter volume loss. **(B)** Sagittal FLAIR of Twin A shows beginning of rarefication of the white matter. **(C)** T2 weighted axial scan of Twin B shows extensive signal abnormalities in the cerebral white matter, with sparing of the subcortical white matter. **(D)** Sagittal FLAIR of Twin B indicates rarefication, however limited in extent.

MRI scans were performed on the twins (Figure 2), which showed findings compatible with VWM. Both twins had extensive cerebral white matter abnormalities, however only Twin A showed significant volume loss. Based on the clinic findings, MRI scan and genetic analysis the diagnosis of VWM was concluded for both**.**

### Genetics

Based on the clinical phenotype Whole-Genome Sequencing (WGS) was performed on DNA from the patient and compared with data from both parents, in order to elucidate whether a genetic explanation could be established. Analyses of the data identified compound heterozygosity for two missense variants in the *EIF2B3* gene, which encodes the gamma subunit of the eIF2B (eIF2Bγ). The variant classification is based on the ACMG guideline (8). It should be noted that the parents were not related and neither had any familial history of congenital neurological diseases.

#### Variant 1: c.260c>T (p.Ala87Val)

This paternally inherited variant was identified in both twin brothers. It has repeatedly been classified as pathogenic or likely pathogenic by reputable ClinVar submitters (variation ID 4439) and is associated with VWM phenotypes. Based on current evidence, we classified this variant as pathogenic (Table 1).

**Table 1:** Classification of the variants according to ACMG guidelines (8). GnomeAD version 4.1 was used to determined variation frequency (9).

Position (GRCh38)	Variant	gnomAD 4.1 Freq (het, hmz)	Applied ACMG criteria	Classification
chr1:44978349	NM_020365.5:c.260C>T;	0.0159% (256/0)	PP1, PP3, PP4, PP5, PM1, PM2	Pathogenic
	NP_065098.1:p.Ala87Val			
chr1:44874807	NM_020365.5:c.1073T>C;	0.00031% (5/0)	PP1, PP3, PP4, PM1, PM2, PM3	Likely pathogenic
	NP_065098.1:p.Ile358Thr			

#### Variant 2: c.1073t>C (p.Ile358Thr)

This maternally inherited missense variant, which has not been described in literature or ClinVar, was identified in both twin brothers. Although previously undescribed, several lines of evidence suggest that this variant has deleterious impact on the protein structure and function.

**Figure 3:**
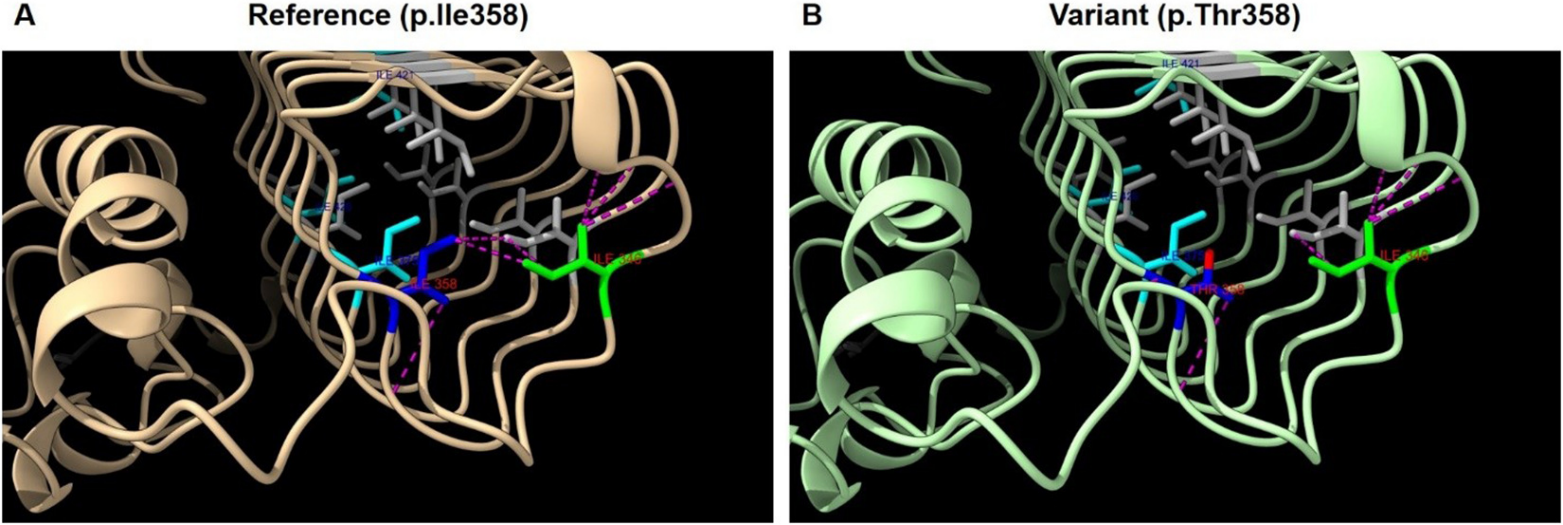
Structural analysis of eIF2Bγ protein model AF-Q9NR50-F1 (AlphaFold). **(A)** Structure of wild-type eIF2Bγ I-patch domain. **(B)** Predicted structure with the p.Ile358Thr variant. Legend: Blue; p.Ile358, Lime; p.Ile346, Cyan; p.Ile375, p.Ile421, p.Ile426, Gray; other isoleucines in the I-patch. Purple dashed lines; predicted contacts involving p.Ile346 and p.Ile358 in **(A)** and p.Thr358 in **(B)**, Red: oxygen atom of Thr358.

Structural modeling using Rotamers in ChimeraX (10) indicates that residue Ile358 is part of a hydrophobic core within the I-patch of eIF2Bγ, where it interacts with neighboring residues such as Ile346 and Ile364 (Figure 3A). Substitution with threonine at the position introduces a polar side chain into this hydrophobic environment, representing a significant physicochemical shift (Figure 3B). The Grantham distance for this change is 89, indicating a moderate-to-high functional impact.

*In silico* modeling using MIZTLI predicts a strongly negative ΔG value (>−2.0 kcal/mol), consistent with significant protein destabilization.

Based on the aforementioned structural predictions, Variant 2 (c.1073T>C) was classified as likely pathogenic according to ACMG guidelines (8) (Table 1).

## Discussion

In this case, Twin A presented with nonspecific symptoms, which were under investigation. The disease phenotype only became apparent during an episode of acute motor function loss after a minor head trauma, which is one of the defining features of VWM disease. The following MRI imaging and genetic analysis verified the clinical diagnosis.

Twin B, carrying the same genetic variant in *EIF2B3* as his symptomatic brother, also showed MRI changes consistent with VWM. The MRI supports that for Twin B the disease could still be in an early phase, which could explain why he was still presymptomatic.

Literature supports that there is a spectrum of clinical manifestations of VWM (5). Regarding age-of-onset, no statistical significant genotype-phenotype correlation has been observed regarding pathogenic variations in the genes for the five subunits (*EIF2B1* to *EIF2B5*). However, within the same genotype age-of-onset and severity appears to be rather consistent. For the pathogenic variants associated with a milder phenotype, some variability in phenotype has been observed (6, 7).

Regarding intrafamilial phenotypes, disease course and severity among siblings appear to be relatively similar, especially with age-of-onset <2 years of age. Adult onset VWM shows wider individual variation. Intrafamilial phenotypic heterogeneity has been reported, suggesting that environmental factors such as fever episodes, sickness and minor head trauma also influence the phenotype (3, 7, 11).

This case could indicate that factors other than genotype could influence age-of-onset. Even though the twins were born on the same day, with the same variants, Twin A had shown symptoms since one year of age, while Twin B still did not show symptoms at the time of the MRI. It should be noted, that according to the parents, Twin A experienced more frequent and prolonged episodes of sickness than his siblings did.

As Twin B does not show marked symptoms of the disease at this time, we can only speculate on age-of-onset.

The patients presented in this case study are compound heterozygote for variants in the *EIF2B3*, a gene implicated in ∼7% of all described VWM cases (12).

The paternally inherited c.260C>T (p.Ala87Val) variant is well-established as pathogenic in the literature. In contrast, the maternally inherited c.1073T>C (p.Ile358Thr) variant has not previously been described. Structural analysis, however, strongly supports a deleterious effect on the protein. It affects the highly conserved left-handed *β*-helix (LbH) domain—referred to as the I-patch—critical for stabilizing the eIF2B holocomplex (11). This domain contains a cluster of hydrophobic isoleucine residues essential for proper protein folding and inter-subunit interactions.

Variants affecting nearby residues (e.g., p.Ile346Thr, p.Ile375Ser, p.Ile421Phe, p.Ile426Thr) have previously been classified as pathogenic and are known to impair holocomplex assembly (13–15), further emphasizing the functional importance of this region. The replacement of a hydrophobic isoleucine with a polar threonine at position 358 likely disrupts critical hydrophobic interactions, compromising domain stability and consistent with established VWM pathogenesis (16).

In this case report we describe two four-year-old twins with VWM and different phenotypes. Twin A had shown unspecific symptoms since one year of age, but was only diagnosed after hospitalization due to minor head trauma. Twin B underwent genetic testing and MRI after Twin A's diagnosis. It was observed that although Twin B was still presymptomatic, he had the same genetic variants as Twin A and MRI findings compatible with VWM. As Twin A had shown symptoms since one year of age, and Twin B had not shown VWM symptoms at the time of MRI, this case may indicate that environmental factors could also affect the age of onset in VWM disease (7).

The twins were compound heterozygote in the *EIF2B3*-gene. One of the variants was not previously described. When compared with the clinical findings and the result of the brain MRI, together with later analysis of the structure and its predicted effect on the function of the protein, the new variant was classified as likely pathogenic using the AMCG classification.

The twins are being closely followed by the pediatric department and preventive measures are implemented in their day-to-day life, such as wearing helmets, vaccinations, together with antibiotics and fever reducing treatment during infections. We are also closely monitoring Twin B for onset of symptoms.

Increased knowledge of the genetic variants in VWM and the different clinical manifestations will hopefully lead to an earlier diagnosis of future VWM patients, and thereby the opportunity for early implementation of preventive measures to avoid episodes of acute worsening of the disease.

## Data Availability

The datasets presented in this study can be found in online repositories. The names of the repository/repositories and accession number(s) can be found in the article/Supplementary Material.

## References

[1] van der Knaap MS Bonkowsky JL Vanderver A Schiffmann R Krägeloh-Mann I Bertini E Therapy trial design in vanishing white matter. Neurology Genetics. (2022) 8(2):e657. 10.1212/NXG.0000000000000657 35128050 PMC8811717

[2] Soderholm HE Chapin AB Bayrak-Toydemir P Bonkowsky JL. Elevated leukodystrophy incidence predicted from genomics databases. Pediatr Neurol. (2020) 111:66–9. 10.1016/j.pediatrneurol.2020.06.005 32951664 PMC7506144

[3] van der Knaap MS Pronk JC Scheper GC. Vanishing white matter disease. Lancet Neurol. (2006) 5(5):413–23. 10.1016/S1474-4422(06)70440-9 16632312

[4] Bugiani M Vuong C Breur M van der Knaap MS. Vanishing white matter: a leukodystrophy due to astrocytic dysfunction. Brain Pathol. (2018) 28(3):408–21. 10.1111/bpa.12606 29740943 PMC8028328

[5] Pronk JC van Kollenburg B Scheper GC van der Knaap MS. Vanishing white matter disease: a review with focus on its genetics. Ment Retard Dev Disabil Res Rev. (2006) 12(2):123–8. 10.1002/mrdd.20104 16807905

[6] van der Knaap MS Fogli A Boespflug-Tanguy O Abbink TEM Schiffmann R. Childhood Ataxia with Central Nervous System Hypomyelination/Vanishing White Matter. In: Adam MP Feldman J Mirzaa GM Pagon RA Wallace SE Amemiya A, editors. GeneReviews®. (online). Seattle, WA: University of Washington (2003). Available online at: https://www.ncbi.nlm.nih.gov/books/NBK1258/ (Accessed July 08, 2025). 20301435

[7] Hamilton EMC van der Lei HDW Vermeulen G Gerver JAM Lourenço CM Naidu S Natural history of vanishing white matter. Ann Neurol. (2018) 84(2):274–88. 10.1002/ana.25287 30014503 PMC6175238

[8] Richards S Aziz N Bale S Bick D Das S Gastier-Foster J Standards and guidelines for the interpretation of sequence variants: a joint consensus recommendation of the American college of medical genetics and genomics and the association for molecular pathology. Genet Med. (2015) 17(5):405–24. 10.1038/gim.2015.30 25741868 PMC4544753

[9] Genome Aggregation Database. (Version 4.1 April 2024) (2014).

[10] Meng EC Goddard TD Pettersen EF Couch GS Pearson ZJ Morris JH UCSF Chimerax: tools for structure building and analysis. Protein Sci. (2023) 32(11):e4792. 10.1002/pro.4792 37774136 PMC10588335

[11] van der Lei HD van Berkel CGM van Wieringen WN Brenner C Feigenbaum A Mercimek-Mahmutoglu S Genotype-phenotype correlation in vanishing white matter disease. Neurology. (2010) 75(17):1555–9. 10.1212/WNL.0b013e3181f962ae 20975056

[12] Scali O Perri CD Federico A. The spectrum of mutations for the diagnosis of vanishing white matter disease. Neurol Sci. (2006) 27(4):271–7. 10.1007/s10072-006-0683-y 16998732

[13] Wang Y Zhang X Zhang H Lu Y Huang H Dong X Coiled-coil networking shapes cell molecular machinery. Mol Biol Cell. (2012) 23(19):3911–22. 10.1091/mbc.e12-05-0396 22875988 PMC3459866

[14] Slynko I Nguyen S Hamilton EMC Wisse LE de Esch IJP de Graaf C Vanishing white matter: eukaryotic initiation factor 2B model and the impact of missense mutations. Mol Genet Genomic Med. (2021) 9(3):e1593. 10.1002/mgg3.1593 33432707 PMC8104162

[15] Lawrence RE Shoemaker SR Deal A Sangwan S Anand AA Wang L A helical fulcrum in eIF2B coordinates allosteric regulation of stress signaling. Nat Chem Biol. (2024) 20(4):422–31. 10.1038/s41589-023-01453-9 37945896 PMC10972756

[16] Wong YL LeBon L Edalji R Lim HB Sun C Sidrauski C. The small molecule ISRIB rescues the stability and activity of vanishing white matter disease eIF2B mutant complexes. Elife. (2018) 7:e32733. 10.7554/eLife.32733 29489452 PMC5829914

